# A Blend Strategy to Achieve High Gain and Long-Term Stability in Complementary Inverters via Vertical Organic Electrochemical Transistors

**DOI:** 10.1021/acsami.5c23663

**Published:** 2026-04-08

**Authors:** Marcos Luginieski, Henrique Frulani de Paula Barbosa, Andreas Schander, Gregório Couto Faria, Björn Lüssem

**Affiliations:** † São Carlos Institute of Physics, 9168 University of São Paulo, São Carlos, São Paulo 13560-970, Brazil; ‡ Institute for Microsensors, Actuators and Systems, 441161 University of Bremen, Bremen 28359, Germany; § MAPEX - Center for Materials and Process, University of Bremen, Bremen 28359, Germany

**Keywords:** Organic electrochemical transistors, Complementary inverter, High gain, p-type blend, Long-term stability

## Abstract

While complementary inverters form the foundation of modern digital electronics, the performance of flexible and wearable counterparts is still limited. Organic electrochemical transistors (OECTs) offer a new route to address this challenge. They can leverage their unique intrinsic ion-to-electron transduction mechanism and high intrinsic transconductance to increase the gain of inverters. Furthermore, the ionic modulation observed in OECTs enables new functionalities, in particular neuromorphic behavior. In this work, complementary inverters based on vertical OECTs (vOECTs) are successfully fabricated, employing poly­(benzimidazobenzophenanthroline) (BBL) as the n-type semiconductor, while poly­(3-hexylthiophene-2,5-diyl) (P3HT), poly­(3-[2-(2-methoxyethoxy)­ethoxy]­ethylthiophene-2,5-diyl) (P3MEEET), or a blend of both polymers is used as p-type counterparts. These devices achieve high voltage gain and fast transient response depending on the employed p-type material system. A maximum voltage gain of 200 V/V is obtained using P3HT-based devices with reduced solution concentration, while P3MEEET-based inverters exhibit faster switching kinetics, with transient response times down to 1 ms. Furthermore, P3HT-based inverters provide superior operational stability when compared with P3MEEET-based devices. By blending both polymers, a balanced device response is achieved, combining the fast transient characteristics of P3MEEET with the stability of P3HT. The resulting blend-based inverters maintain stable operation for over 18,000 cycles at 10 Hz, demonstrating a versatile vOECT platform and an effective materials-engineering approach to simultaneously achieve high speed, high gain, and robust long-term stability in organic electronics.

## Graphical abstract

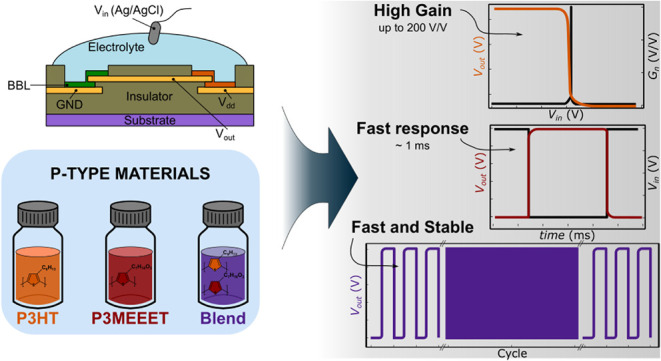

## Introduction

The logic inverter is a fundamental cornerstone of modern electronics.^1^ They enable basic logical operations through two stable electrical logic levels by inverting the input signal in a step function-like response. Thus, logic inverters serve as a building block for data processing, storage, and the construction of more complex logic gates. Beyond logic operations, inverters can also be applied as voltage amplifiers, where small input signals near the switching threshold induce large output changes. This is an essential property for oscillators, buffers, and timing circuits.

Among different designs, the complementary inverter has become the standard due to its efficiency and scalability. Built from an n-type and a p-type transistor connected in series, the complementary metal-oxide-semiconductor (CMOS) inverter ensures that one transistor is ON while the other is OFF, minimizing power consumption. This complementary operation delivers high gain, fast switching, and robust noise margins, making CMOS inverters fundamental in processors, memories, and integrated systems.

In the field of organic transistors, complementary inverters based on organic electrochemical transistors (OECTs) have been primarily employed as sensors for electrolyte concentration and as amplifiers in neuromorphic circuits.^2–9^ In sensing applications, the most critical parameter is the shift in the inversion voltage (the point of maximum gain) with electrolyte concentration, rather than the absolute maximum gain value.^6^ In amplifier applications, such as neuromorphic voltage amplifier blocks, inverters must deliver high voltage gain, fast response, and a stable inversion voltage.^5,9^ Therefore, the development of complementary OECT inverters with high gain, rapid switching, and stable operation is of great importance.

Over time, complementary OECT inverters have achieved remarkable improvements in both gain and dynamic performance. One of the earliest demonstrations, in 2018, presented a fully organic complementary inverter using depletion-mode OECTs based on BBL (n-type) and P3CPT (p-type) semiconductors, achieving a gain of approximately 12 V/V.^2^ By 2022, printed complementary OECT circuits reached significantly higher performance: a single-stage inverter achieved gains up to 26 V/V at a supply voltage below 0.7 V, while a two-stage configuration delivered a record 193 V/V gain, one of the highest among emerging CMOS-like technologies operating at sub-1 V.^3^ This work also emphasized ultralow power consumption and optimized amplifier design for sensitive detection.

A major leap in performance came in 2023 with the introduction of vertical OECT (vOECT) architecture. Using a vertically stacked structure, Huang et al. demonstrated gains of approximately 150 V/V at a 0.7 V supply.^6^ These devices also featured ultrastable switching over more than 30,000 cycles at a frequency of 10 Hz. More recently, Yao et al. reported a high-performance vOECT-based inverter achieving transient response times below 1 ms, combined with an exceptionally high voltage gain exceeding 400 V/V, surpassing previous vertical designs, and more than 50,000 reliable switching cycles.^9^ Despite these impressive demonstrations, the design remains limited by material constraints, as the devices were developed for specific organic semiconductors.

In this work, we explore the fabrication of complementary inverters based on step-edge vOECTs that combine high gain, fast transient response, and stability, while allowing greater flexibility in semiconductor selection. The main focus of this work is to tune the performance of complementary inverters based on step-edge vOECTs. Specifically, the goal is to achieve high voltage gain, fast switching speed, and long-term operational stability. Our vOECT-based inverter design enables the on-demand choice and deposition of semiconductor layers. As the n-type semiconductor, we use the well-known poly­(benzimidazobenzophenanthroline) (BBL) polymer, while the p-type counterpart is based on poly­(3-hexylthiophene-2,5-diyl) (P3HT), poly­(3-[2-(2-methoxyethoxy)­ethoxy]­ethylthiophene-2,5-diyl) (P3MEEET), or a blend of both polymers. The inverters based on BBL/P3MEEET polymers exhibit a gain of 112 V/V and switching speeds in the order of 1 ms. BBL/P3HT, on the other hand, demonstrates superior stability, and by changing the p-type polymer concentration, we were able to tune the maximum gain up to 200 V/V. Finally, the blend-based inverters demonstrate switching speeds on the order of a few milliseconds and stability over more than 18,000 cycles. Our work demonstrates new strategies and tools to improve complementary inverters’ performance.

## Results and Discussion

Complementary inverters consist of two transistors of opposite polarity connected in series, as shown in the circuit diagram in Figure 1a. Following our previous work,^10^ the step-edge vertical OECT (vOECT) architecture is selected as the technological platform for the inverter. As illustrated in Figure 1b, the two bottom gold electrodes serve as the ground and supply voltage contacts, while the top gold electrode is shared by both transistors and works as the output contact. Details of the fabrication process are provided in the Experimental Section.

**Figure 1:**
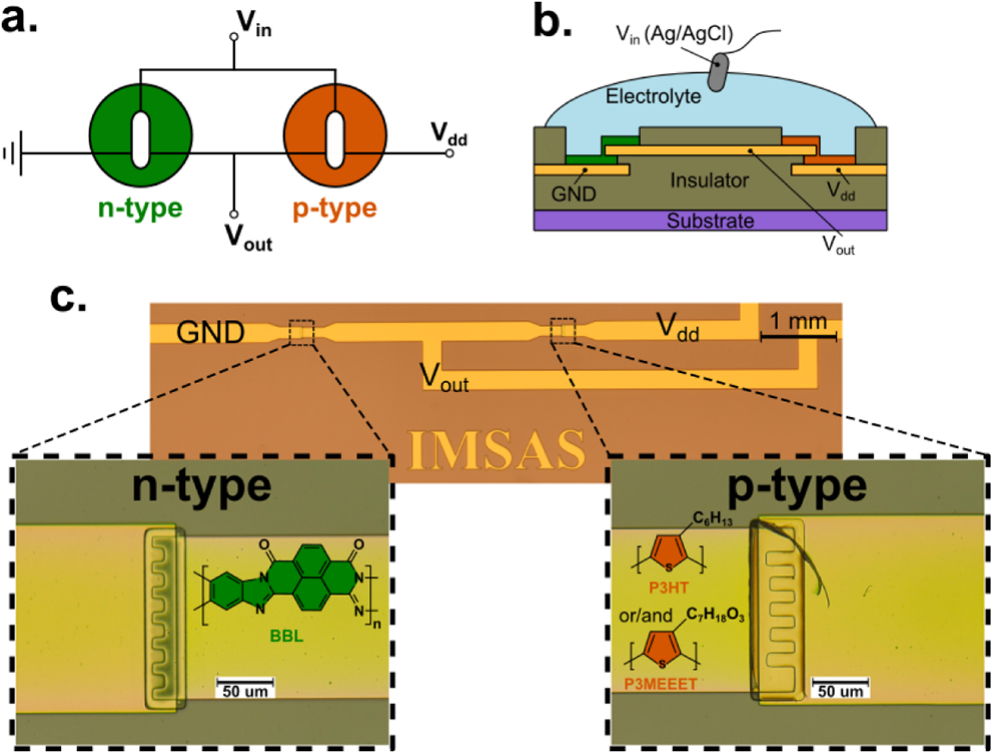
**a.** Circuit diagram of the complementary inverter. **b.** Side-view illustration of the vOECTs used in the inverter. **c.** Microscopic image of the inverter substrate (*W*_n‑type_ = 300 μm and *W*_p‑type_ = 400 μm), with chemical structures of the organic semiconductors used.

Each semiconductor layer is deposited individually by spin-coating, using a physical barrier placed at the center of the substrate to prevent cross-contamination. First, the n-type semiconductor is deposited and subsequently annealed. The p-type semiconductor is then deposited on the remaining half of the substrate and annealed in sequence. Finally, a sacrificial polyimide layer is peeled off to define the channel openings. A single external Ag/AgCl pellet, immersed in the electrolyte covering both transistors, is used as the common gate/input electrode.

Figure 1c presents a microscopy image of one inverter substrate, showing both n-type and p-type transistors in detail. Although fabrication requires a physical barrier during spin coating, this method provides the advantage of on-demand semiconductor deposition. Moreover, the step-edge design enables high transconductance within a compact footprint area.^10^

Throughout this work, the n-type polymer will remain unchanged. The individual vOECT performance of each material is shown in the (Supporting Information see Figures S1–S4). The p-type material is varied to realize BBL/P3MEEET and BBL/P3HT inverters. The P3HT devices, however, are fabricated using two different concentrations: 20 and 10 mg/mL, hereafter referred to as P3HT-Hi and P3HT-Lo, respectively. These devices are classified as nonblend-based inverters, as they are fabricated using a single p-type semiconductor. To further tune the inverter performance, particularly the switching speed and operational stability, a blend of both p-type materials is introduced, resulting in the BBL/Blend inverter, hereafter referred to as the blend-based device.

### Nonblend-Based Devices

Figure 2a summarizes the voltage-transfer-characteristics (VTCs) curves for each inverter at *V*_dd_ = 0.7 V. The full set of VTCs is provided in the Supporting Information (see Figures S5–S7). The maximum voltage gain (*G*_n_) obtained for each inverter is shown in Figure 2b.

**Figure 2:**
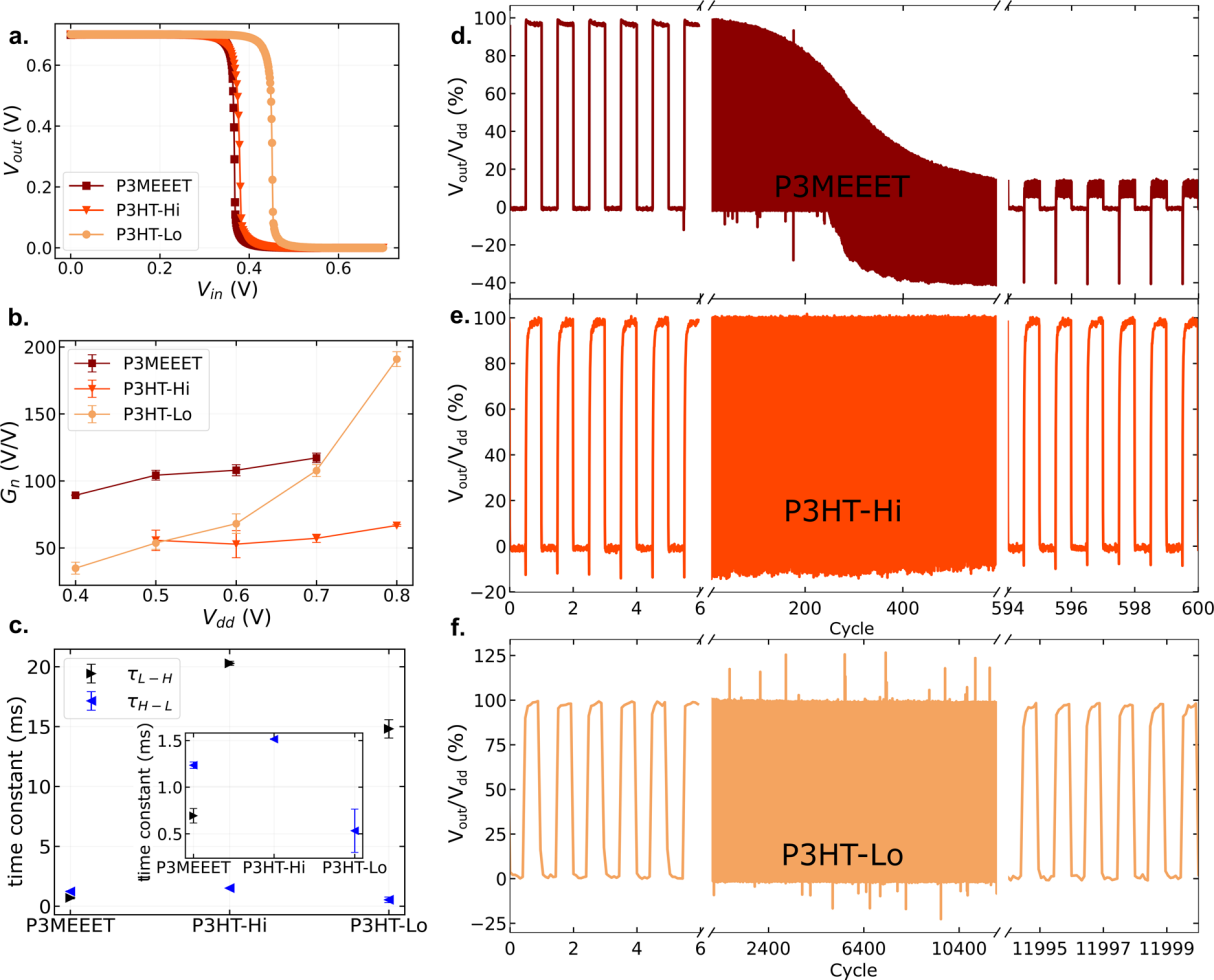
**a.** VTCs of each device at *V*_dd_ = 0.7 V. **b.** Maximum gain for each *V*_dd_ tested. **c.** High-to-low (τ_H–L_) and low-to-high (τ_L–H_) time constants for each inverter. The inset highlights the τ_H–L_. **d**–**f** Normalized stability test for the three inverters.

The BBL/P3MEEET devices exhibit a maximum gain of approximately 90 V/V at *V*_dd_ = 0.4 V, which increases to 122 V/V at a supply voltage of 0.7 V. This performance is comparable to that of state-of-the-art complementary OECT inverters.^3,4,6,8,11,12^ In contrast, the BBL/P3HT-Hi inverters, despite exhibiting larger maximum transconductance in the individual vOECTs, show a relatively lower and nearly constant voltage gain of around 60 V/V across all tested supply voltages. Finally, the BBL/P3HT-Lo inverter achieves the highest overall gain, reaching approximately 200 V/V at *V*_dd_ = 0.8 V, in line with recent reported vOECT-based complementary inverters.^6,7,9,13–15^ A table comparing the gain across different devices is provided in the Supporting Information (see Table S1). Additionally, the gain vs frequency response of BBL/P3HT-Lo inverter can be found in Figure S8.

To better understand these results, it is instructive to revisit the definition of the voltage gain (*G*_n_) in complementary inverters. The voltage gain is defined as

1 $$G_{n}={\frac{\partial V_{\mathrm{out}}}{\partial V_{\mathrm{in}}}|}_{V_{\mathrm{dd}}}$$

and can be further expressed in terms of the intrinsic parameters of the individual transistors as

2 $$G_{n}=-\frac{g_{m,n}+g_{m,p}}{g_{d,n}+g_{d,p}}$$

where $g_{m}=\frac{dI_{\mathrm{DS}}}{dV_{\mathrm{GS}}}$ and $g_{d}=\frac{dI_{\mathrm{DS}}}{dV_{\mathrm{DS}}}$ represent the transconductance and output conductance, respectively, of the n-type and p-type transistors. According to eq 2, high-gain inverters are obtained by combining large transconductance values with low output conductance in the saturation regime of both transistors. Therefore, it is essential to achieve a nearly constant saturation current in each transistor to maximize the inverter voltage gain.

Indeed, BBL, P3MEEET, and P3HT-Lo devices exhibit *g*_d_ ∼ 0.1 mS, whereas P3HT-Hi shows a significantly higher value of *g*_d_ ∼ 10 mS (see Figures S1–S4), which explains that P3MEEET and P3HT-Lo devices perform better than the higher-concentration P3HT counterparts. The difference between the two P3HT cases arises from the lower drain currents of the P3HT-Lo devices. The lower semiconductor concentration of P3HT results in decreased conductivity and residual conductivity of the channel.^16^ This is a consequence of a less crystalline or less densely packed film^17^ which in turn reduces the intrinsic charge-carrier mobility and consequently decreases σ. This behavior may be associated, for instance, with a decrease in the number of percolation paths available for charge transport, which effectively lowers the carrier mobility.^18^ Therefore, minimizing the saturation *g*_d_ constitutes a crucial strategy for achieving high-gain inverters, as it directly governs the inverter gain.

In terms of switching speed, two characteristic time constants are defined for inverters: the high-to-low transition (τ_H–L_), determined by the turn-ON of the n-type device and the turn-OFF of the p-type device, and the low-to-high transition (τ_L–H_), determined by the turn-OFF of the n-type device and the turn-ON of the p-type device. As shown in Figure 2c, τ_H–L_ is on the order of 1 ms for all devices, which is attributed to the fast turn-ON response of the BBL transistors.^4,19,20^ On the other hand, the BBL/P3MEEET inverters exhibit a faster τ_L–H_ compared to both P3HT-based cases. This improvement is attributed to the enhanced ionic diffusion in P3MEEET arising from its glycolated side chains^21,22^ which facilitates faster electrochemical kinetics.

Despite their promising performance, the BBL/P3MEEET inverters display poor operational stability during long-term pulsing tests, as shown in Figure 2d. After 100 pulsing cycles, at a frequency of 1 Hz, a small degradation in the high output level is already observed, and after 500 cycles, the inverter becomes completely nonoperational. It is worth noting that the high logic level corresponds to the state where the p-type transistor is ON and the n-type transistor is OFF; therefore, the degradation is directly associated with the P3MEEET-based transistor. Although the origin of this instability requires further investigation, it may be related to electrochemical side reactions or structural degradation of the polymer under repeated gating and has already been reported in literature.^10,23^

In contrast, both P3HT-based inverters exhibit excellent operational stability. The P3HT-Hi inverter withstands more than 600 pulsing cycles, at a frequency of 1 Hz, without noticeable loss in either logic level, while the P3HT-Lo device remains stable for over 12,000 cycles (10 Hz).

### Blend-Based Devices

From the previous results, it is concluded that, despite their poor stability, P3MEEET-based devices exhibit fast switching, whereas P3HT-based devices show excellent long-term stability but slower kinetics. To further improve the inverter performance, a blend of these two p-type materials is investigated. A 1:1 polymer mixture is prepared by dissolving 10 mg of P3HT and 10 mg of P3MEEET in 1 mL of chlorobenzene, yielding a final concentration of 20 mg/mL. All subsequent fabrication steps for the p-type devices are carried out as previously described. This strategy of combining two semiconductor polymers has already been explored in the literature.^24–27^ In particular, P3MEEET-fullerene^26^ and P3HT-fullerene^27^ blends have been investigated to achieve stable and balanced ambipolar operation in OECTs. To the best of our knowledge, however, the present work is the first to investigate a blend of two structurally similar polymers to enhance the overall p-type performance.

The steady-state performance of the blend-based vOECTs is presented in Figure 3a–d. The devices exhibit efficient current modulation and negligible hysteresis. The maximum transconductance reaches 30 mS at *V*_DS_ = −0.4 V, while the saturation conductance is on the order of 1 mS. These characteristics closely resemble those of the P3MEEET-based devices, particularly the low hysteresis observed in the transfer curves. Since hysteresis is typically associated with charge-transport kinetics and the doping/dedoping dynamics^28^ this behavior already suggests that the blend-based OECTs retain the fast-switching characteristics of P3MEEET.

**Figure 3:**
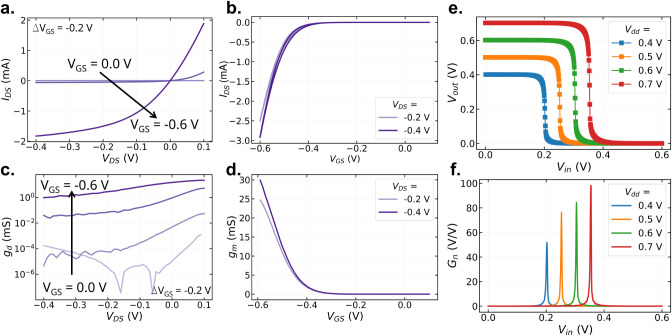
**a.** Output and **b.** transfer curves, **c.** conductance, and **d.** transconductance of blend-based vOECTs. **e.** VTC and **f.** gain of BBL/Blend inverters. For both cases *W*_BBL_ = 300 μm and *W*_Blend_ = 400 μm.

The blend is subsequently implemented in complementary inverters using BBL as the n-type semiconductor. As shown in Figure 3e and f, the resulting devices exhibit a well-behaved voltage transfer characteristics (VTCs), with maximum gains increasing from 51.8 V/V at *V*_dd_ = 0.4 to 98.1 V/V at *V*_dd_ V. Although the gain is lower than that achieved with the optimized single-material inverters, it remains comparable to other OECT-based complementary inverters reported in the literature.^3,4,8^ The frequency-dependent gain response of this device can be seen in Figure S9.

To address the central question, whether the blend enables both fast and stable inverter operation, the transient response was evaluated by pulsing the input voltage at fixed frequencies while maintaining a constant supply voltage. The inverters were tested across frequencies ranging from 1 to 200 Hz. As shown in Figure 4a–b, the devices exhibit reliable switching up to 200 Hz, with time constants on the order of 1 ms, consistent with the performance of BBL/P3MEEET inverters. Notably, 200 Hz represents the average upper limit before degradation of the high-level output is observed; however, some devices operated up to 600 Hz with only a 40% reduction in the high-level signal (see Figure S10). Finally, both time constants decrease with increasing frequency. This behavior can be attributed to incomplete ionic equilibration and the frequency-dependent effective capacitance of the OECT channels. The effective capacitance of both transistors decreases with frequency, leading to shorter time constants.

**Figure 4:**
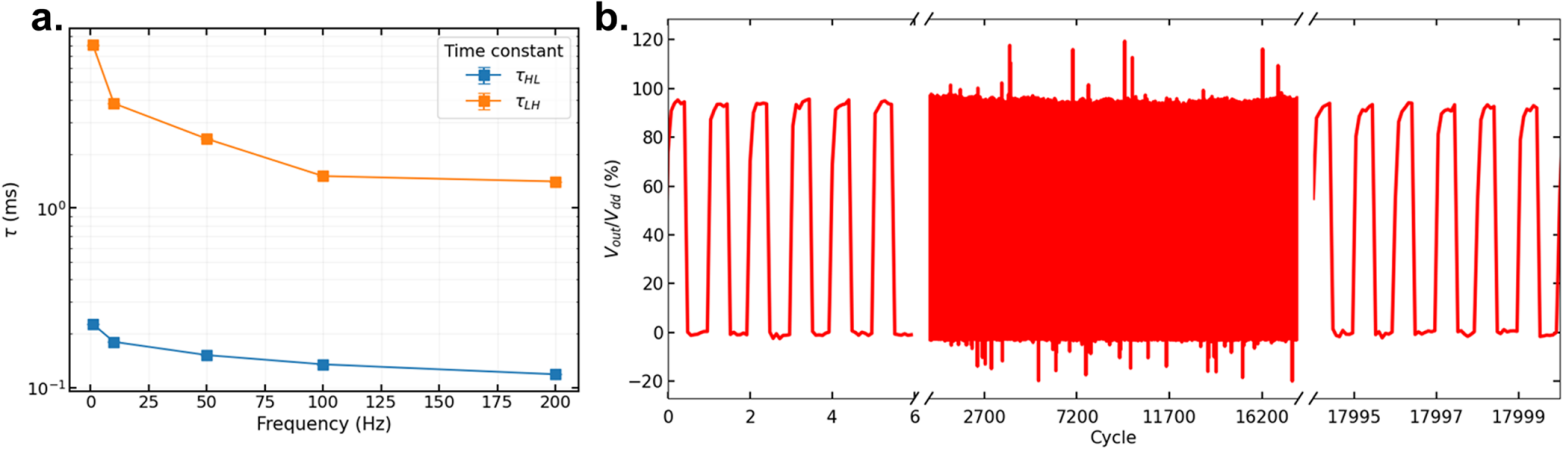
**a.** Time constant of the transient response at different frequencies. **b.** Normalized stability test for *V*_in_ = 0.6 V at a frequency of 10 Hz.

The operational stability of the inverters is further evaluated at a fixed frequency of 10 Hz. As shown in Figure 4a–b, the devices maintain stable switching for over 18,000 cycles without any detectable degradation of either logic level. This long-term stability is attributed to the presence of P3HT domains within the blend, which contributes to improved stability robustness.

Although a more detailed chemical and morphological investigation is required to fully elucidate the behavior of the polymer blend, the present results suggest that P3MEEET domains facilitate rapid ion penetration and extraction, thereby governing the overall device kinetics and enabling fast switching, whereas P3HT domains provide stable charge modulation over time, mitigating the degradation typically observed in P3MEEET-only devices. Consequently, while P3MEEET contributes to the fast doping dynamics, the presence of P3HT ensures long-term operational stability, leading to a balance between speed and durability.

### Morphological Investigation

To better understand the electrical performance of the inverters, Atomic Force Microscopy (AFM) and X-ray Diffraction (XRD) are used to investigate the surface morphology of each material. Figure 5 depicts the AFM phase images of each film and the corresponding diffraction spectra.

**Figure 5:**
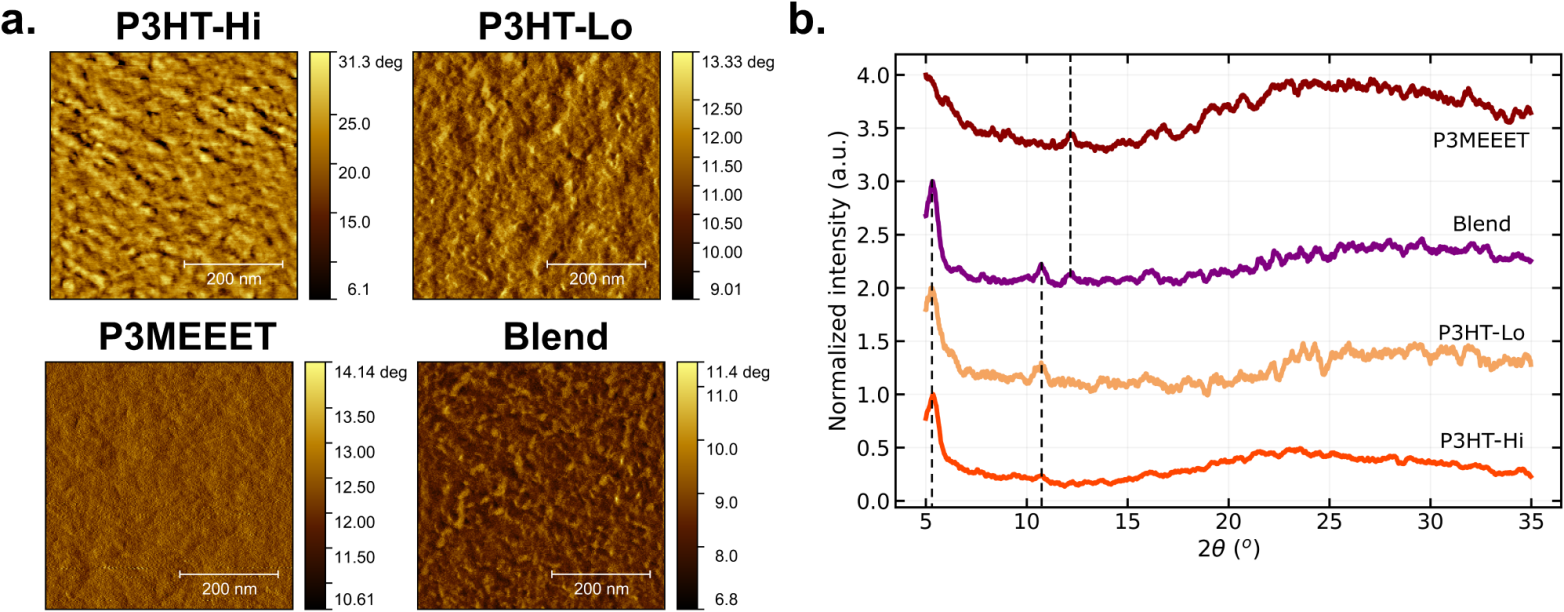
**a.** AFM phase images of all films coated on a gold substrate. **b.** Normalized XRD response of the same samples. The dashed lines indicate common peaks.

From the AFM analysis, comparison of the phase images of the two P3HT samples reveals distinct topologies, which can be associated with differences in morphology. In particular, the P3HT-Hi sample exhibits a higher grain density than the P3HT-Lo sample. These grains may be associated with polymer nanofibers, potentially originating from preaggregation in solution that remained after deposition or from crystallization during the annealing process. As a result, enhanced electronic mobility may be expected, since a higher fiber density is typically correlated with improved transistor performance.^29^ This observation is consistent with the electrical performance of the P3HT-based OECTs. Although a definitive conclusion regarding film crystallinity cannot be drawn solely from AFM, the lower grain density in P3HT-Lo may indicate a more amorphous morphology. The P3MEEET sample shows no clear evidence of fibrous structures and presents a very smooth topology, suggesting a predominantly amorphous morphology. Finally, the blend phase combines the lower grain density observed in P3HT-Lo with a smooth background attributed to the P3MEEET phase.

From the XRD results shown in Figure 5b, the diffraction patterns of all films can be observed. Both P3HT samples and the blend exhibit a sharp peak around 5.2°, attributed to lamellar stacking of the polymer chains, indicative of crystalline ordering.^21,30,31^ For P3HT-Lo, however, this peak is broader than for P3HT-Hi, suggesting a lower degree of crystallinity. The P3MEEET film shows a peak shifted toward lower angles, partially outside the measured range. Additionally, a peak at 10.6° is observed for both P3HT samples, while a peak at 12.2° appears for the P3MEEET film. These features are also present in the blend film. Finally, all samples exhibit a broad shoulder at angles above 15°.

Taken together, the AFM and XRD results support our hypothesis that P3HT-Lo is less crystalline than P3HT-Hi and that the blend film contains phase-separated domains associated with the two different polymers.

### Conclusions

In this work, we present high-performance complementary inverters based on the step-edge vertical organic electrochemical transistor architecture, employing BBL as the n-type semiconductor and P3HT, P3MEEET, or a blend of both as the p-type counterpart. The inverters exhibit high voltage gain and fast switching speeds. P3MEEET-based devices display rapid switching due to the presence of glycolated side chains but suffer from poor operational stability. In contrast, P3HT-based devices demonstrate excellent stability, maintaining performance over more than 12,000 cycles, and, by tuning the solution concentration, achieve the highest voltage gain of approximately 200 V/V. Finally, by blending P3MEEET and P3HT, fast and stable inverter operation is realized. Overall, this work establishes a versatile step-edge vOECT platform and demonstrates an effective polymer-blend strategy that balances high gain, fast transient response, and outstanding long-term operational stability in organic complementary inverters. Nevertheless, further chemical and morphological investigations are needed to fully elucidate the mechanisms underlying the observed performance.

## Experimental Section

### Materials Preparation

The polymers poly­(3-hexylthiophene) M̅_w_ = 50–70 kDa and poly­(3-[2-[2-(2-methoxyethoxy)­ethoxy]­ethyl]­thiophene-2,5-diyl) *M*_w_ = 0.23 kDa, P3HT and P3MEEET, respectively, were purchased from Rieke Metals. Poly­(benzimidazobenzophenanthroline) (BBL), methanesulfonic acid 99.0% (MSA), and lithium bis­(trifluoromethanesulfonyl)­imide (LiTFSI) were purchased from Sigma-Aldrich Co. Chlorobenzene was purchased from TCI. All materials were used as received.

The n-type BBL is dissolved in MSA at a concentration of 5 mg/mL, stirred at 70 °C overnight. Both P3MEEET and P3HT-Hi are dissolved in chlorobenzene at a concentration of 20 mg/mL and stirred at 60 °C overnight. P3HT-Lo is dissolved in chlorobenzene at a concentration of 10 mg/mL, following the same stirring procedure. The blend is prepared by mixing 10 mg of P3HT and 10 mg of P3MEEET in 1 mL of chlorobenzene. The solution is stirred overnight at 60 °C. All material preparation is made in open-air conditions.

### vOECT/Inverter Fabrication

The fabrication of the inverter chip follows the microfabrication steps as described previously in refs 10, 32 .

In the IMSAS ISO-6 cleanroom, 4 in. silicon/500 nm thick silicon oxide layer wafers are spin-coated with a 5 μm thick polyimide layer (U-Varnish S, UBE Corporation) and then cured using a vacuum hot plate. A 250 nm thick gold layer is then deposited by magnetron DC sputtering (Pro Line PVD 75, Kurt J. Lesker Company Ltd.). Then, photolithography (AZ 1518, MicroChemicals GmbH) and wet chemical etching (Au etch 200, NB Technologies GmbH) are used to structure the bottom electrode layer (ground and supply voltage electrodes). On top, a 300 nm insulating polyimide layer is spin-coated and cured, defining the channel length of the transistors. Sequentially, the second gold layer is deposited following the same process as before. Again, photolithography and wet chemical etching are used to structure the top electrodes (output voltage contact). After this, a short (30 s) oxygen RIE plasma treatment (STS ICP) is performed, and a 300 nm thick polyimide layer is spin-coated and cured. Another 5 μm-thick polyimide layer is spin-coated and cured. This additional layer is the sacrificial layer to define the channels. All polyimide layers are structured by photolithography (AZ 10XT, MicroChemicals GmbH) and oxygen RIE plasma (STS ICP). The wafers are then diced and are ready to use.

Before semiconductor solution deposition, a physical barrier is positioned in the middle of the chip to avoid cross-contamination of the transistors. First, BBL is spin-coated at 1000 rpm for 60 s. The chip is then immersed in ethanol for 1 min. Afterward, a light blow of nitrogen is used to dry the excess of ethanol. Next, the films are annealed at 150 °C for 5 min. Finally, the p-type solution is spin-coated at 2000 rpm for 60 s and then annealed at 120 °C for 1 h. All p-type devices are prepared following the same steps. The LiTFSI electrolyte is prepared at a concentration of 100 mM in DI water. The gate/input electrode is an external Ag/AgCl pellet.

### vOECT/Inverter Electrical Characterization

Initially, the steady-state transfer and output curves of each transistor are measured using a Keithley 2612b. Then, the inverters VTC are recorded using the Keithley 2612b (applies *V*_in_ and measures *V*_out_) and a function generator Agilent 33522A (applies *V*_dd_). For these measurements, *V*_dd_ is ranged from 0.4 to 0.8 V with steps of 0.1 V, while *V*_in_ is ranged from 0 to 0.7 V, with steps of 1 mV.

Transient measurements and stability tests are performed using the dual-channel function generator Agilent 33522A to apply *V*_in_ pulses and the constant *V*_dd_ (0.6 V). The *V*_in_ consisted of a square wave alternating between 0 and 0.6 V, with a 50% duty cycle. The pulse frequency was adjusted as required. The output voltage is recorded in a Tektronix MSO46 Oscilloscope.

The steady-state measurements are carried out using the SweepMe software. All data processing and plotting are done with custom Python codes.

### Atomic Force Microscopy (AFM)

The topography measurements were performed in tapping mode with a Nanoscope III AFM microscope, using a SiN_3_ probe. The data was analyzed and edited with Gwyddion open source software.

### X-ray Diffraction (XRD)

The crystalline structure of the thin films was investigated by XRD from the Laboratory of Nanomaterials and Advanced Ceramics (NaCA) from São Carlos Institute of Physics (IFSC-USP), using a Rigaku Ultima IV diffractometer, Cu Kα source, range of 5–80°, at angular steps of 0.02° and 3 s per point of acquisition time. Gold is used as the internal standard.

## Supplementary Material



## Data Availability

The data that support the findings of this study are available from the corresponding author upon reasonable request.

## Notes

Steady-state performance of each vOECT; steady-state, transient response, and long-term stability test of each inverter; gain vs frequency response; transient response tests of the blend-based inverter; table summarizing several OECT-based inverters (PDF)
