# Supplementary material for: A Blend Strategy to Achieve High Gain and Long-Term Stability in Complementary Inverters via Vertical Organic Electrochemical Transistors
Source: ACS Appl Mater Interfaces. 2026 Apr 8;18(15):22193–200. doi: 10.1021/acsami.5c23663 (PMC13107373; doi:10.1021/acsami.5c23663)
Supplement: Supplementary file 1 [file am5c23663_si_001.pdf]

Supporting Information: A Blend Strategy to Achieve High Gain and Long-Term Stability in Complementary Inverters via Vertical Organic Electrochemical Transistors

Marcos Luginieski,*,†,‡,¶ Henrique Frulani de Paula Barbosa,‡ Andreas Schander,‡ Gregório Couto Faria,*,† and Björn Lüssem*,‡,¶

†*São Carlos Institute of Physics, University of São Paulo, PO Box 369, 13560-970, São Carlos, SP, Brazil*

‡*Institute for Microsensors, Actuators and Systems, University of Bremen, 28359, Bremen, Germany*

¶*MAPEX - Center for Materials and Process University of Bremen, 28359, Bremen, Germany*

E-mail: mluginieski@ifsc.usp.br; gcfaria@ifsc.usp.br; bluessem@uni-bremen.de

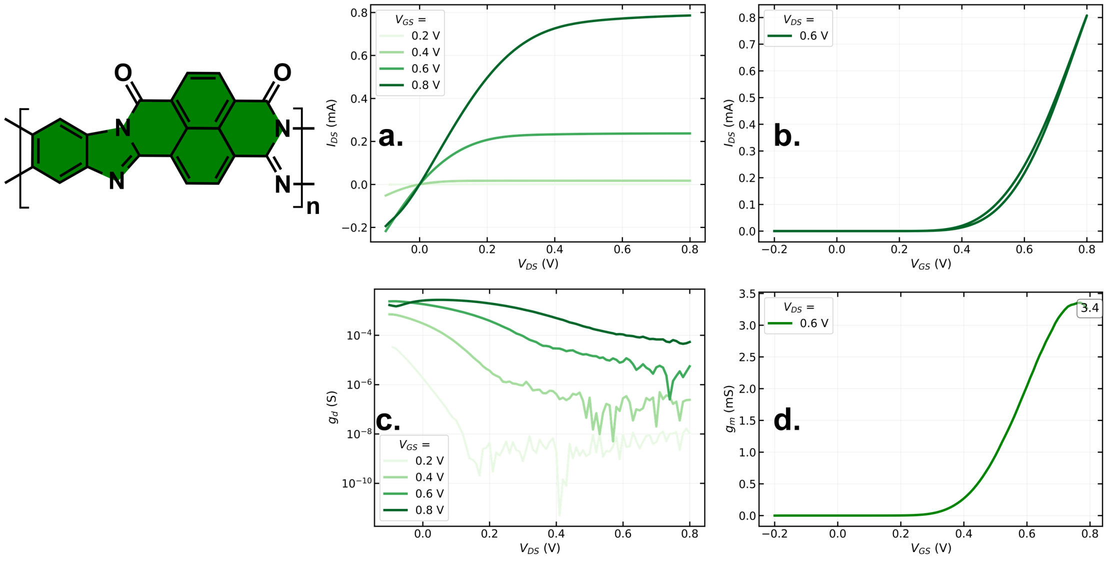

Figure S1: **a.** Output, **b.** transfer, **c.** channel conductance and **d.** maximum transconductance of BBL vOECT, where $W_{\mathrm{BBL}} = 300\ \mu$m. The electrolyte is 100 mM LiTFSI and the gate electrode is a Ag/AgCl pellet.

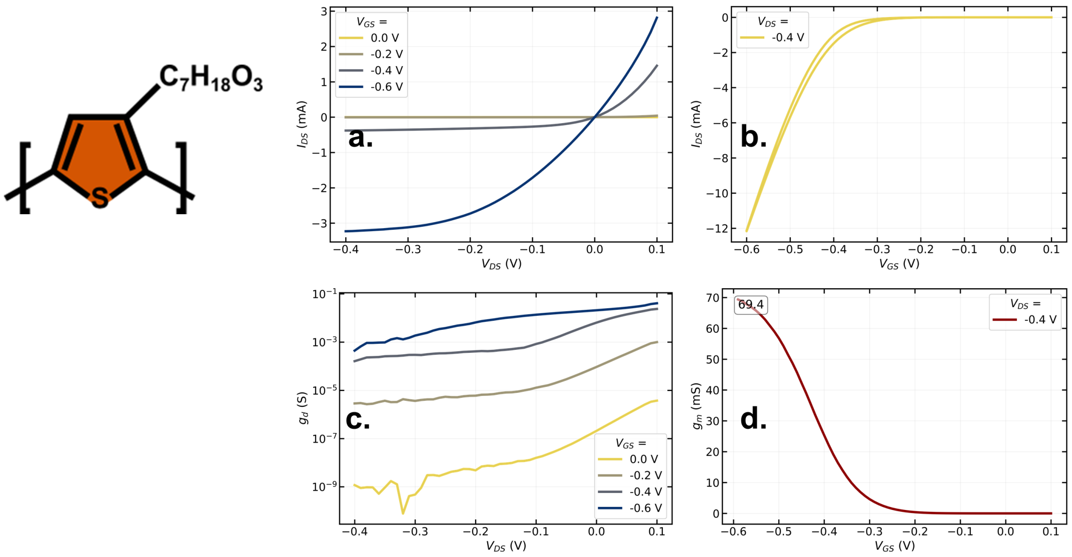

Figure S2: **a.** Output, **b.** transfer, **c.** channel conductance and **d.** maximum transconductance of P3MEEET vOECT, where $W_{\mathrm{P3MEEET}} = 400\ \mu$m. The electrolyte is 100 mM LiTFSI and the gate electrode is a Ag/AgCl pellet.

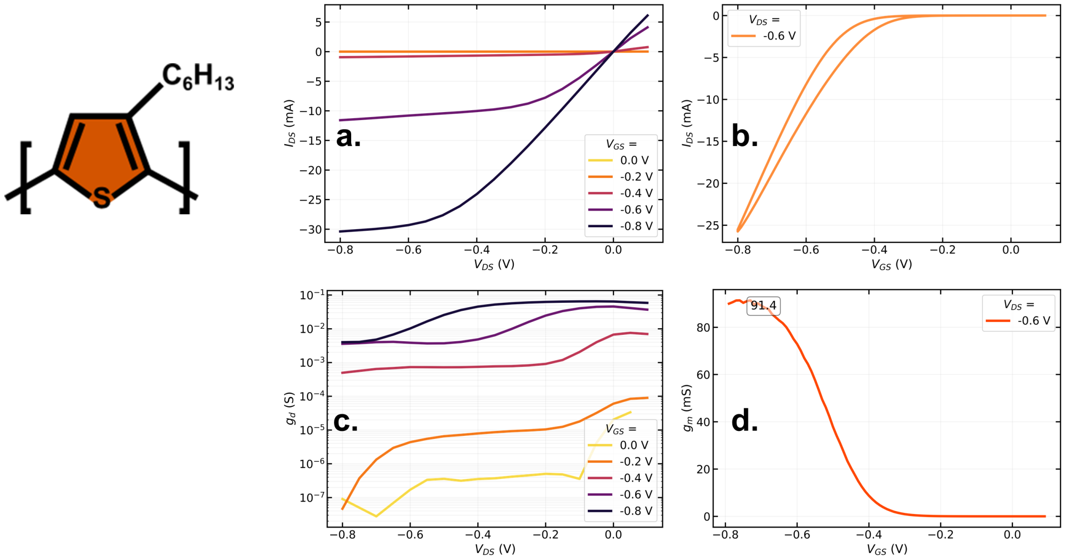

Figure S3: **a.** Output, **b.** transfer, **c.** channel conductance and **d.** maximum transconductance of P3HT-Hi vOECT, where $W_{\mathrm{P3HT}} = 400\ \mu$m. The electrolyte is 100 mM LiTFSI and the gate electrode is a Ag/AgCl pellet.

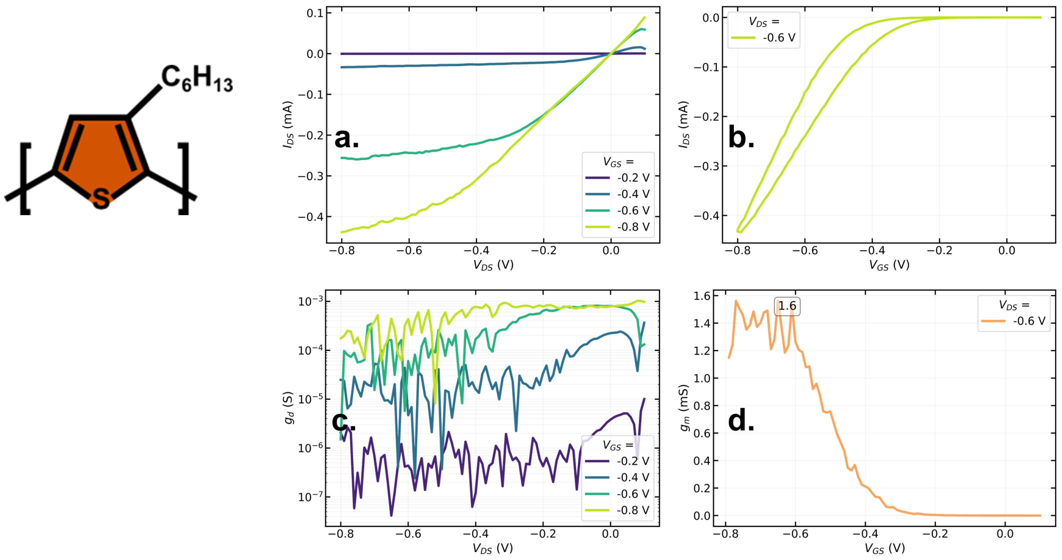

Figure S4: **a.** Output, **b.** transfer, **c.** channel conductance and **d.** maximum transconductance of P3HT-Lo vOECT, where $W_{\mathrm{P3HT}} = 400\ \mu$m. The electrolyte is 100 mM LiTFSI and the gate electrode is a Ag/AgCl pellet.

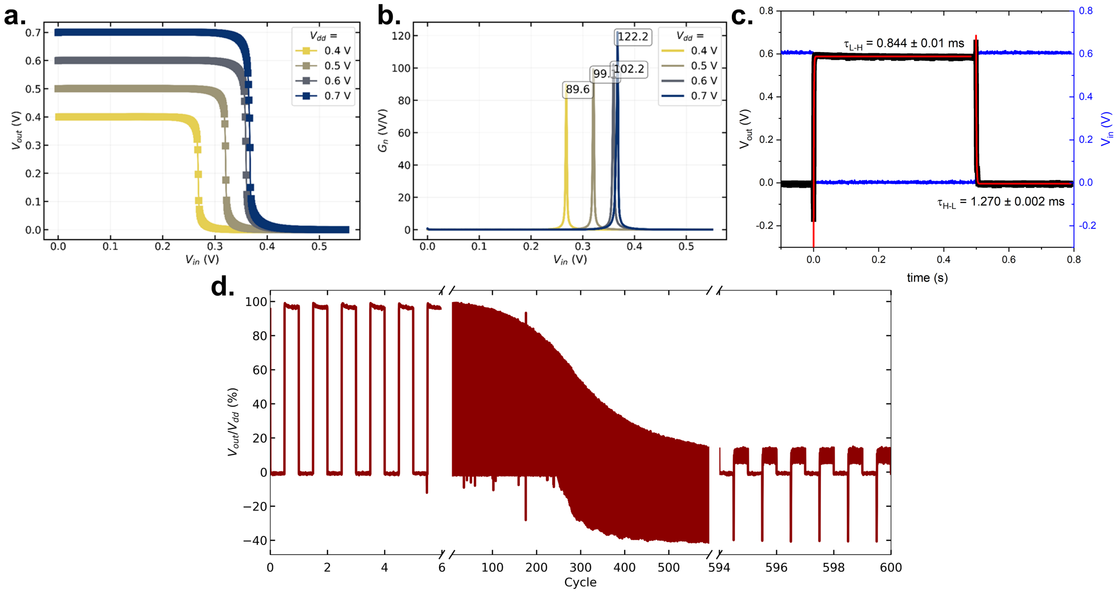

Figure S5: **a)** VTC, **b)** voltage gain, **c)** transient response and **d)** stability of BBL/P3MEEET inverters at 1 Hz. In the transient response of the output voltage (black line), the fitting (red line) is done with and exponential decay function. Here $W_{\mathrm{BBL}} = 300$ μm and $W_{\mathrm{P3MEEET}} = 400$ μm.

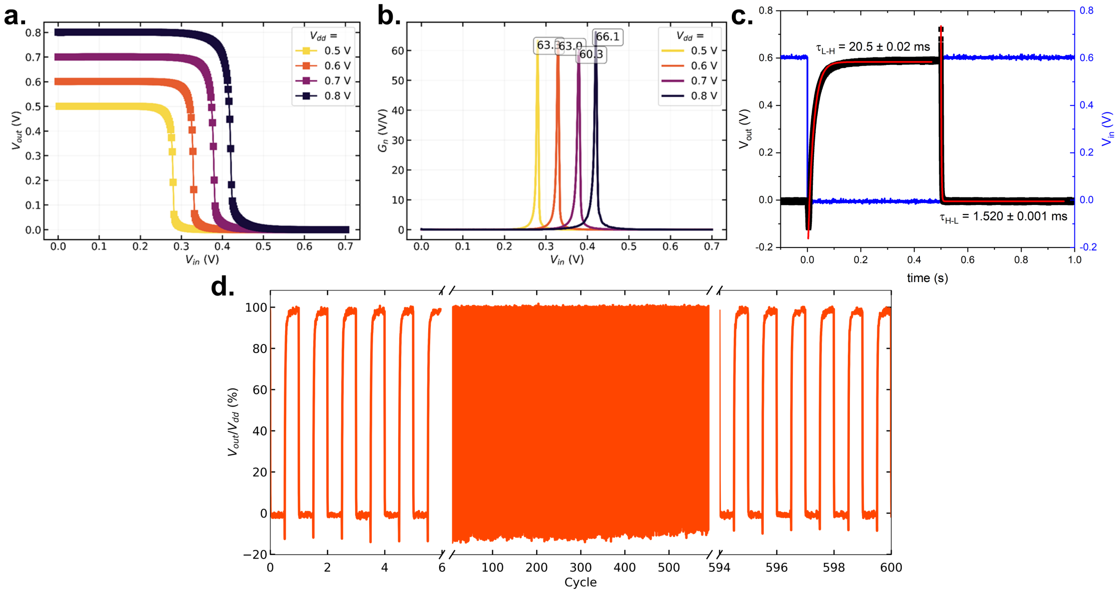

Figure S6: **a)** VTC, **b)** voltage gain, **c)** transient response and **d)** stability of BBL/P3HT inverters. In the transient response of the output voltage (black line), the fitting (red line) is done with and exponential decay function. Here $W_{\mathrm{BBL}} = 300$ μm and $W_{\mathrm{P3HT}} = 400$ μm.

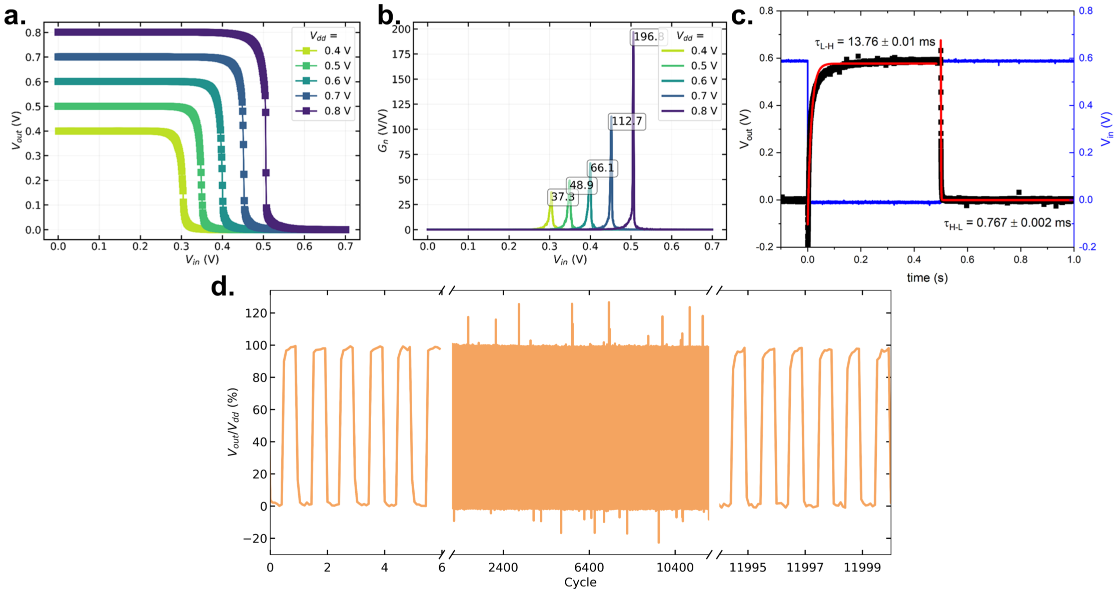

Figure S7: **a)** VTC and **b)** gain of BBL/P3HT (10 mg/ml) inverters. **c)** Conductance and **d)** transconductance of P3HT vOECT measured after inverter characterization. Again $W_{\text{BBL}} = 300\ \mu$m and $W_{\text{P3HT}} = 400\ \mu$m

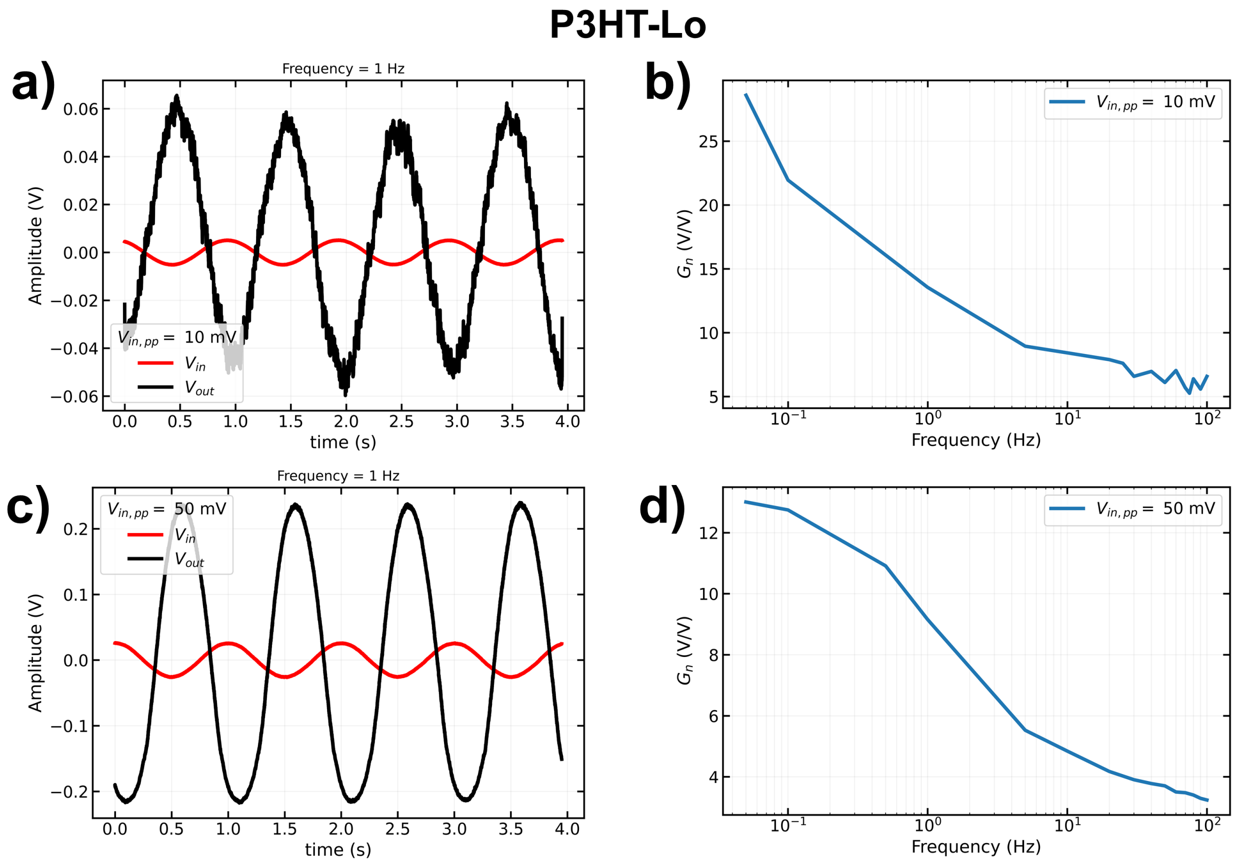

Figure S8: **a)** Voltage amplification of the P3HT-Lo inverter and **b)** frequency-dependent gain for 10 mV. **c)** Voltage amplification and **d)** frequency-dependent gain for 50 mV.

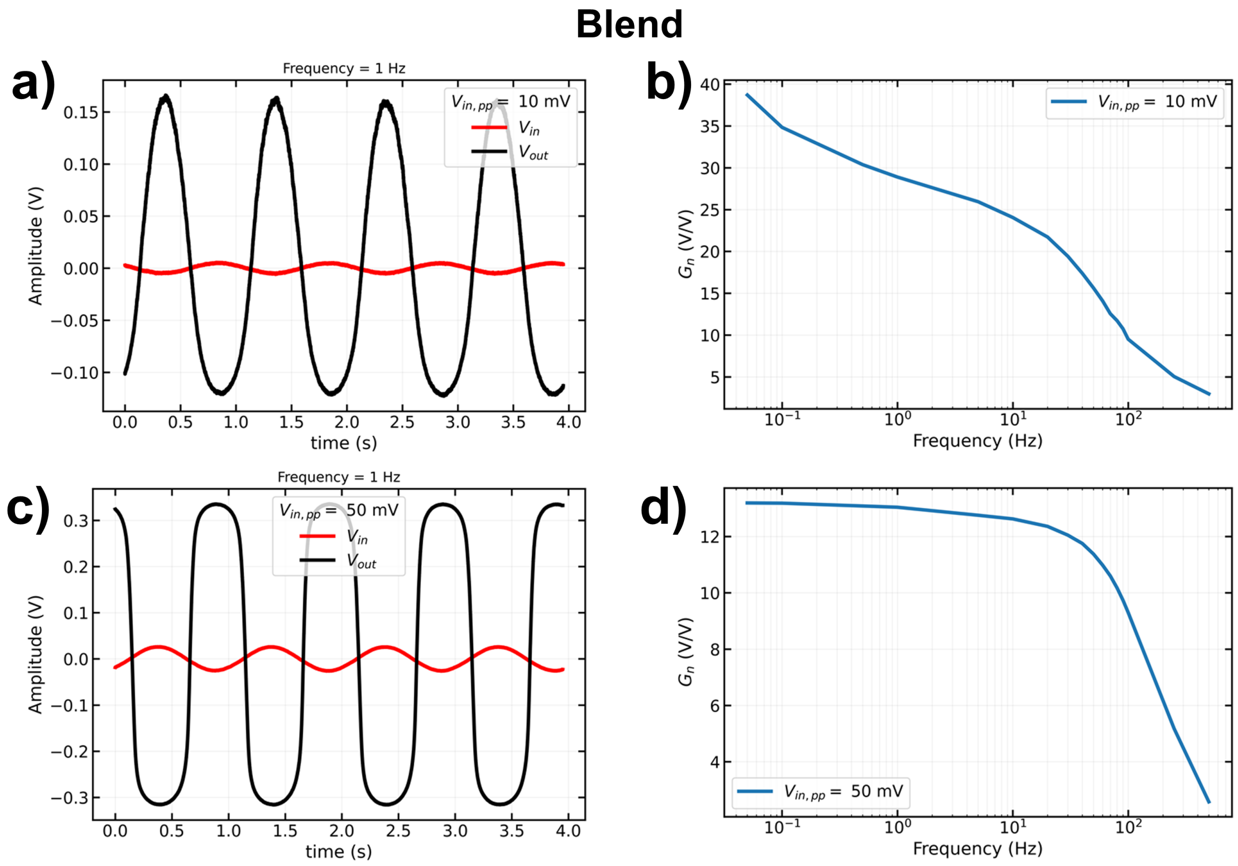

Figure S9: **a)** Voltage amplification of the blend-based inverter and **b)** frequency-dependent gain for 10 mV. **c)** Voltage amplification and **d)** frequency-dependent gain for 50 mV.

The gain values extracted from the VTC curves and those obtained via the frequency-dependent method are not equivalent, likely due to asymmetric transient effects and the fundamentally different nature of the gain extraction approaches. Such discrepancies are commonly reported in the literature[S1,S2]

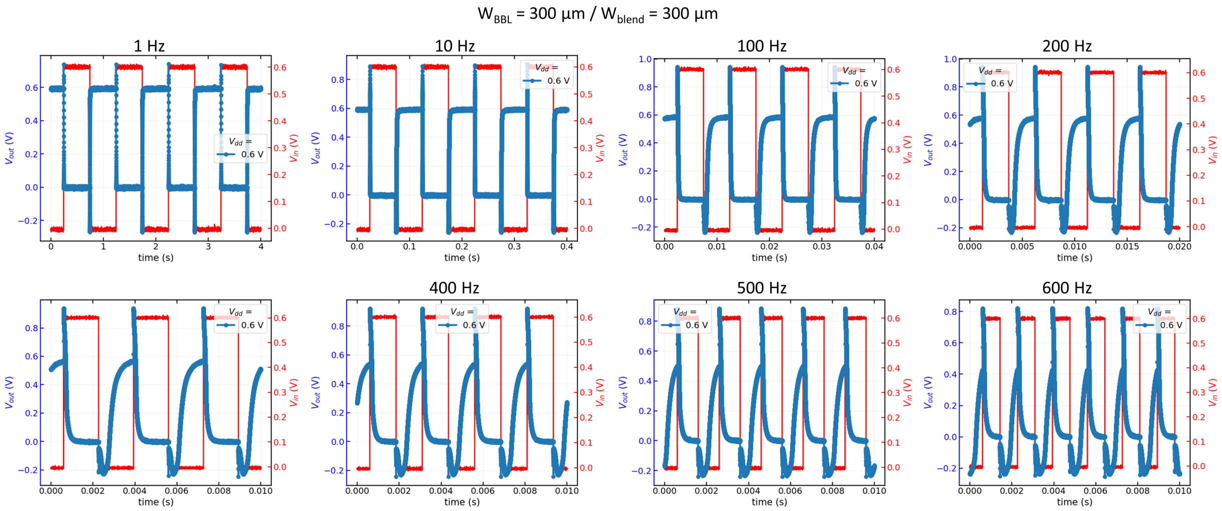

Figure S10: Transient response of one BBL/Blend inverter at different frequencies.

Table S1: Summary of Organic semiconductor-based inverters.

Channel	Type	OSC	V_{dd}	Gain	Stability, Freq.	Ref.
Planar	ambipolar	PThDPP-BTz	1.3	101	-	[S3]
Planar	ambipolar	PThDPP-BBTz	0.8	93	-	[S3]
Planar	ambipolar	DHF-gTT	0.8	102	-	[S4]
Planar	ambipolar	DH-gTT	0.8	31.7	-	[S4]
Planar	blend/ambipolar	PrC_60MA:p(g2T-TT)	0.9	82.56	20, -	[S5]
Planar	BHJ/ambipolar	BBL:PBBTL	-0.6	42	-	[S6]
Planar	BHJ/ambipolar	BBL:PBBTL	0.6	31	-	[S6]
Planar	complementary	BBL/P3CPT	0.6	$\tilde{1}1$	-	[S7]
Planar	complementary	BBL/P($g_4$2T-T)	0.7	26	-	[S8]
Planar	complementary	BBL_{152}/P($g_4$2T-T)	0.7	$\tilde{1}00$	-	[S9]
Planar	complementary	n-PT3/Pg2T-TT	0.9	92.3	-	[S10]
Planar	complementary	n-PT3/Pg2Tz-T	0.8	307	50, 0.5	[S10]
Planar	complementary	BBL/P3CPT	0.7	151	-	[S11]
Planar	complementary	Cl_2-BAL/p(g1T2-g5T2)	0.5	35	5000,	[S12]
Planar	complementary	BBL/P3HT	0.7	46	-	[S13]
Vertical	ambipolar	p(gDPP-V)	0.8	105	10000, 10	[S14]
Vertical	ambipolar	p(C_4-T2-C_0-EG)	0.8	28	24, 0.1	[S1]
Vertical	complementary	BBL/gDPP-g2T	0.6	42.23	10000, 10	[S15]
Vertical	complementary	Homo-gDPP/gDPP-g2T	0.7	150	30000, 10	[S16]
Vertical	complementary	Homo-gDPPTz/gDPP-g2T	0.7	433	50000, 100	[S2]
**Vertical**	**complementary**	**BBL/P3MEEET**	**0.7**	**122**	**>100, 1**	**This work**
**Vertical**	**complementary**	**BBL/P3HT-Hi**	**0.8**	**66**	**600, 1**	**This work**
**Vertical**	**complementary**	**BBL/P3HT-Lo**	**0.8**	**197**	**12000, 10**	**This work**
**Vertical**	**blend/complementary**	**BBL/P3MEEET:P3HT**	**0.7**	**98**	**18000, 10**	**This work**
		n/p	(V)	(V/V)	Cycle, (Hz)	

References

(S1) Rashid, R. B.; Du, W.; Griggs, S.; Maria, I. P.; McCulloch, I.; Rivnay, J. Ambipolar inverters based on cofacial vertical organic electrochemical transistor pairs for biosignal amplification. *Science Advances* **2021**, *7*, eabh1055.

(S2) Yao, Y.; Pankow, R. M.; Huang, W.; Wu, C.; Gao, L.; Cho, Y.; Chen, J.; Zhang, D.; Sharma, S.; Liu, X.; Wang, Y.; Peng, B.; Chung, S.; Cho, K.; Fabiano, S.; Ye, Z.; Ping, J.; Marks, T. J.; Facchetti, A. An organic electrochemical neuron for a neuromorphic perception system. *Proceedings of the National Academy of Sciences* **2025**, *122*, e2414879122.

(S3) Shi, L.; Yang, H.; Li, H.; Kuang, Y.; Ma, M.; Shao, S.; Xie, Z.; Liu, J. Controlling ambipolar OECT threshold voltage through acceptor unit engineering of conjugated polymers. *Journal of Materials Chemistry C* **2025**, *13*, 19437–19443.

(S4) Qi, G.; Wang, M.; Wang, S.; Zhang, S.; Teng, X.; Bai, H.; Wang, B.; Zhao, C.; Su, W.; Fan, Q.; Ma, W. High-Performance, Single-Component Ambipolar Organic Electrochemical Transistors with Balanced n/p-Type Properties for Inverter and Biosensor Applications. *Advanced Functional Materials* **2025**, *35*, 2413112.

(S5) Stein, E.; Nahor, O.; Stolov, M.; Freger, V.; Petruta, I. M.; McCulloch, I.; Frey, G. L. Ambipolar blend-based organic electrochemical transistors and inverters. *Nature Communications* **2022**, *13*, 5548.

(S6) Wu, X.; Tam, T. L. D.; Chen, S.; Salim, T.; Zhao, X.; Zhou, Z.; Lin, M.; Xu, J.; Loo, Y.; Leong, W. L. All-Polymer Bulk-Heterojunction Organic Electrochemical Transistors with Balanced Ionic and Electronic Transport. *Advanced Materials* **2022**, *34*.

(S7) Sun, H.; Vagin, M.; Wang, S.; Crispin, X.; Forchheimer, R.; Berggren, M.; Fabiano, S.

Complementary Logic Circuits Based on High-Performance n-Type Organic Electrochemical Transistors. *Advanced Materials* **2018**, *30*, 1704916.

(S8) Yang, C.-Y.; Tu, D.; Ruoko, T.-P.; Gerasimov, J. Y.; Wu, H.-Y.; Harikesh, P. C.; Massetti, M.; Stoeckel, M.-A.; Kroon, R.; Müller, C.; Berggren, M.; Fabiano, S. Low-Power/High-Gain Flexible Complementary Circuits Based on Printed Organic Electrochemical Transistors. *Advanced Electronic Materials* **2022**, *8*, 2100907.

(S9) Wu, H.-Y.; Yang, C.-Y.; Li, Q.; Kolhe, N. B.; Strakosas, X.; Stoeckel, M.-A.; Wu, Z.; Jin, W.; Savvakis, M.; Kroon, R.; Tu, D.; Woo, H. Y.; Berggren, M.; Jenekhe, S. A.; Fabiano, S. Influence of Molecular Weight on the Organic Electrochemical Transistor Performance of Ladder-Type Conjugated Polymers. *Advanced Materials* **2022**, *34*, 2106235.

(S10) Kuang, Y.; Yao, T.; Deng, S.; Dong, J.; Ye, G.; Zhang, L.; Shao, S.; Zhu, Z.; Liu, J.; Liu, J. Matching P- and N-type Organic Electrochemical Transistor Performance Enables a Record High-gain Complementary Inverter. *Advanced Materials* **2025**, *37*, 2417691.

(S11) Tang, C. G.; Wu, R.; Chen, Y.; Zhou, Z.; He, Q.; Li, T.; Wu, X.; Hou, K.; Kousseff, C. J.; McCulloch, I.; Leong, W. L. A Universal Biocompatible and Multifunctional Solid Electrolyte in p-Type and n-Type Organic Electrochemical Transistors for Complementary Circuits and Bioelectronic Interfaces. *Advanced Materials* **2024**, *36*, 2405556.

(S12) Wu, X.; He, Q.; Zhou, Z.; Tam, T. L. D.; Tang, C.; Lin, M.; Moser, M.; Griggs, S.; Marks, A.; Chen, S.; Xu, J.; McCulloch, I.; Leong, W. L. Stable n-Type Perylene Derivative Ladder Polymer with Antiambipolarity for Electrically Reconfigurable Organic Logic Gates. *Advanced Materials* **2024**, *36*.

(S13) Hou, K.; Chen, S.; Moudgil, A.; Wu, X.; Tam, T. L. D.; Lew, W. S.; Leong, W. L. High

Performance, Flexible, and Thermally Stable All-Solid-State Organic Electrochemical Transistor Based on Thermoplastic Polyurethane Ion Gel. *ACS Applied Electronic Materials* **2023**, *5*, 2215–2226.

(S14) Cong, S.; Chen, J.; Xie, M.; Deng, Z.; Chen, C.; Liu, R.; Duan, J.; Zhu, X.; Li, Z.; Cheng, Y.; Huang, W.; McCulloch, I.; Yue, W. Single ambipolar OECT–based inverter with volatility and nonvolatility on demand. *Science Advances* **2024**, *10*, eadq9405.

(S15) Hu, G.; Cai, Q.; Liu, Z.; Zeng, R.; Feng, L.-W.; Chen, J.; Xiahou, S.; Huang, W. Dynamic Modulation of OECT-Based Inverters for In Situ Electrophysiological Monitoring. *Advanced Science n/a*, e12755.

(S16) Huang, W.; Chen, J.; Yao, Y.; Zheng, D.; Ji, X.; Feng, L.-W.; Moore, D.; Glavin, N. R.; Xie, M.; Chen, Y.; Pankow, R. M.; Surendran, A.; Wang, Z.; Xia, Y.; Bai, L.; Rivnay, J.; Ping, J.; Guo, X.; Cheng, Y.; Marks, T. J.; Facchetti, A. Vertical organic electrochemical transistors for complementary circuits. *Nature* **2023**, *613*, 496–502.
